# A machine learning framework for automated diagnosis and computer-assisted planning in plastic and reconstructive surgery

**DOI:** 10.1038/s41598-019-49506-1

**Published:** 2019-09-19

**Authors:** Paul G. M. Knoops, Athanasios Papaioannou, Alessandro Borghi, Richard W. F. Breakey, Alexander T. Wilson, Owase Jeelani, Stefanos Zafeiriou, Derek Steinbacher, Bonnie L. Padwa, David J. Dunaway, Silvia Schievano

**Affiliations:** 1 UCL Great Ormond Street Institute of Child Health, London, UK; 2 Craniofacial Unit, Great Ormond Street Hospital for Children, London, UK; 3 Department of Plastic and Oral Surgery, Boston Children’s Hospital & Harvard School of Dental Medicine, Boston, MA USA; 4 Department of Computing, Imperial College London, London, UK; 5 Department of Plastic and Reconstructive Surgery, Yale University School of Medicine, New Haven, CT USA

**Keywords:** Medical imaging, Translational research, Biomedical engineering

## Abstract

Current computational tools for planning and simulation in plastic and reconstructive surgery lack sufficient precision and are time-consuming, thus resulting in limited adoption. Although computer-assisted surgical planning systems help to improve clinical outcomes, shorten operation time and reduce cost, they are often too complex and require extensive manual input, which ultimately limits their use in doctor-patient communication and clinical decision making. Here, we present the first large-scale clinical 3D morphable model, a machine-learning-based framework involving supervised learning for diagnostics, risk stratification, and treatment simulation. The model, trained and validated with 4,261 faces of healthy volunteers and orthognathic (jaw) surgery patients, diagnoses patients with 95.5% sensitivity and 95.2% specificity, and simulates surgical outcomes with a mean accuracy of 1.1 ± 0.3 mm. We demonstrate how this model could fully-automatically aid diagnosis and provide patient-specific treatment plans from a 3D scan alone, to help efficient clinical decision making and improve clinical understanding of face shape as a marker for primary and secondary surgery.

## Introduction

Over 200,000 maxillofacial procedures, including orthognathic (jaw) surgery, are performed in the USA every year to treat a range of diseases, defects and injuries in the head, neck and face^1^. For these operations, vast quantities of patient data are collected^2^, thus providing a great opportunity for the development of machine-learning-based methods, for use in clinical decision-making and to enable automated personalised medicine approaches^3,4^. Although the application of machine learning in plastic and reconstructive surgery is not new – in orthognathic surgery it has been used to elucidate how syndromes affect skull growth^5^, to quantify^6^ or to predict^7^ the corrective effect of surgical techniques on skull deformities, and for outcome evaluation^8^ – its clinical usefulness has been limited due to the low number of samples, absence of automated processing methods, and lack of state-of-the-art mathematical models. Therefore, we propose a machine-learning-based framework involving a large number of data points and fully automated processing for diagnosis and clinical decision making in orthognathic surgery.

Medical imaging and computer-assisted surgical planning form an integral part of the preoperative workup^9,10^, as exploring various operative approaches in a virtual environment can reduce operation time^11^ and cost^12^, and facilitate more consistent and optimised surgery^13^. A digital patient model, reconstructed from computed tomography (CT) or magnetic resonance (MR) images^14,15^, can be manipulated by the surgeon to determine the optimal bone cuts (osteotomies) and bone position, and to simulate changes in face shape^16,17^. Based on this virtual patient-specific treatment plan, custom-made surgical wafers, cutting guides, plates and implants can be manufactured to deliver the plan^18,19^. Although 3D computer-assisted surgical planning has been around for over 30 years^20^, and there are known benefits over traditional 2D planning^21^, implementation in clinical practice has been limited to highly specialised hospitals, mainly due to the complexity of commercial software^22^ and the contested planning accuracy^23–26^.

Machine-learning-based models, including statistical shape models, have been proposed to streamline and automate processes in computer-assisted surgical planning^27^, thereby making this a more accessible technology. However, accurate statistical modelling of face shape features is a challenging task due to the large anatomical variation in the human population; and to build a statistical model that can truthfully represent each given face, a large collection of high-quality 3D images is required from a population diverse in age, gender, and ethnicity^28–31^. State-of-the-art computer vision algorithms are required to automatically process these 3D images and construct a high-dimensional statistical model. A popular machine learning approach, originally used to reconstruct accurate and complete 3D representations from single 2D images and for photo-realistic manipulation^32^, involves 3D morphable models (3DMM) – statistical models of face shape and texture. Current applications of 3DMM include facial recognition^33^, expression normalisation^34^, and face reconstruction from videos^35^.

Here, we present the first fully-automated large-scale clinical 3DMM involving supervised learning for diagnostics, risk stratification, and treatment simulation. Using databases comprising 10,000 3D face scans of healthy volunteers and patients admitted for orthognathic surgery, we trained and validated a 3DMM, and demonstrated its potential for clinical decision making, including fully-automated diagnosis and surgery simulation. We believe our proposed model is an important step towards making computer-assisted surgical planning cheaper, and more accessible for surgeons and patients. This model could potentially transform patient-specific clinical decision making in orthognathic surgery and other fields of plastic and reconstructive surgery.

## Results

In this section, to demonstrate the power of our large-scale clinical 3DMM, we present the following results: (1) a description of how the models were built and the 3D face databases used, including intrinsic statistical validation metrics; (2) an evaluation of mean face shape to compare, quantitatively and qualitatively, how patient faces differ from volunteer faces preoperatively and postoperatively; (3) a manifold visualisation to compare high-dimensional patient and volunteer shape data; (4) a classification for automated diagnosis of faces with orthognathic shape features as an indication for orthognathic surgery, and (5) an analysis of different regression techniques to simulate patient faces for automated patient-specific surgical planning.

### Model construction and validation

Two databases were used to build the 3DMMs, comprising volunteer and patient 3D face scans. For the volunteer faces, we used the LSFM database (see Methods) which contains 9,663 3D face scans from the general public with mean age 24.5 ± 14.7 years, 52% female, and 82% white heritage (Table 1). For the patient faces, 274 3D scans were retrospectively selected from a database of 151 patients who underwent orthognathic procedures at Boston Children’s Hospital and Yale-New Haven Hospital, with mean age at surgery 18.4 ± 2.4 years, 56% female, and 76% white heritage (Table 1). Additional patient demographics are summarised in Supplementary Fig. 1.

**Table 1:** Characteristics of patient and volunteer faces in the databases.

Characteristics	Dataset		
	Patient	LSFM dataset (all ages)	LSFM subset (14–28 yr)
Number of subjects	151	9,663	3,943
Number of images	273	9,663	3,943
Age: mean (s.d.), years	18.4 (2.4), *n* = 151	24.5 (14.7), *n* = 9,460	22.2 (3.7), *n* = 3,943
Age: range, years	14–28, *n* = 151	0–85, *n* = 9,460	14–28, *n* = 3,943
Gender (% male/female)	44%/56%, *n* = 151	47.7%/52.2%, *n* = 9,582	55.5%/44.5%, *n* = 3,942
Ethnicity	72% White, 10% Asian, 10% Mixed Heritage/Other, 8% Black, *n* = 151	82% White, 9% Asian, 5% Mixed Heritage, 3% Black, 1% Other, *n* = 9,554	83% White, 8% Asian, 4% Mixed Heritage, 3% Black, 2% Other, *n* = 3,928

*n* is the number of individuals for whom that measurement was available. s.d. indicates the standard deviation.

We trained three 3DMM (see Methods): a global model, a bespoke preoperative model, and a bespoke postoperative model. The global model (n = 4,216) comprised all patient scans as well as volunteer scans from the same age range (Table 1). The bespoke preoperative (n = 119) and postoperative (n = 127) models were made exclusively with patient scans – ‘bespoke’ refers to the fact that these models are custom made to represent preoperative or postoperative patient faces.

**Figure 1:**
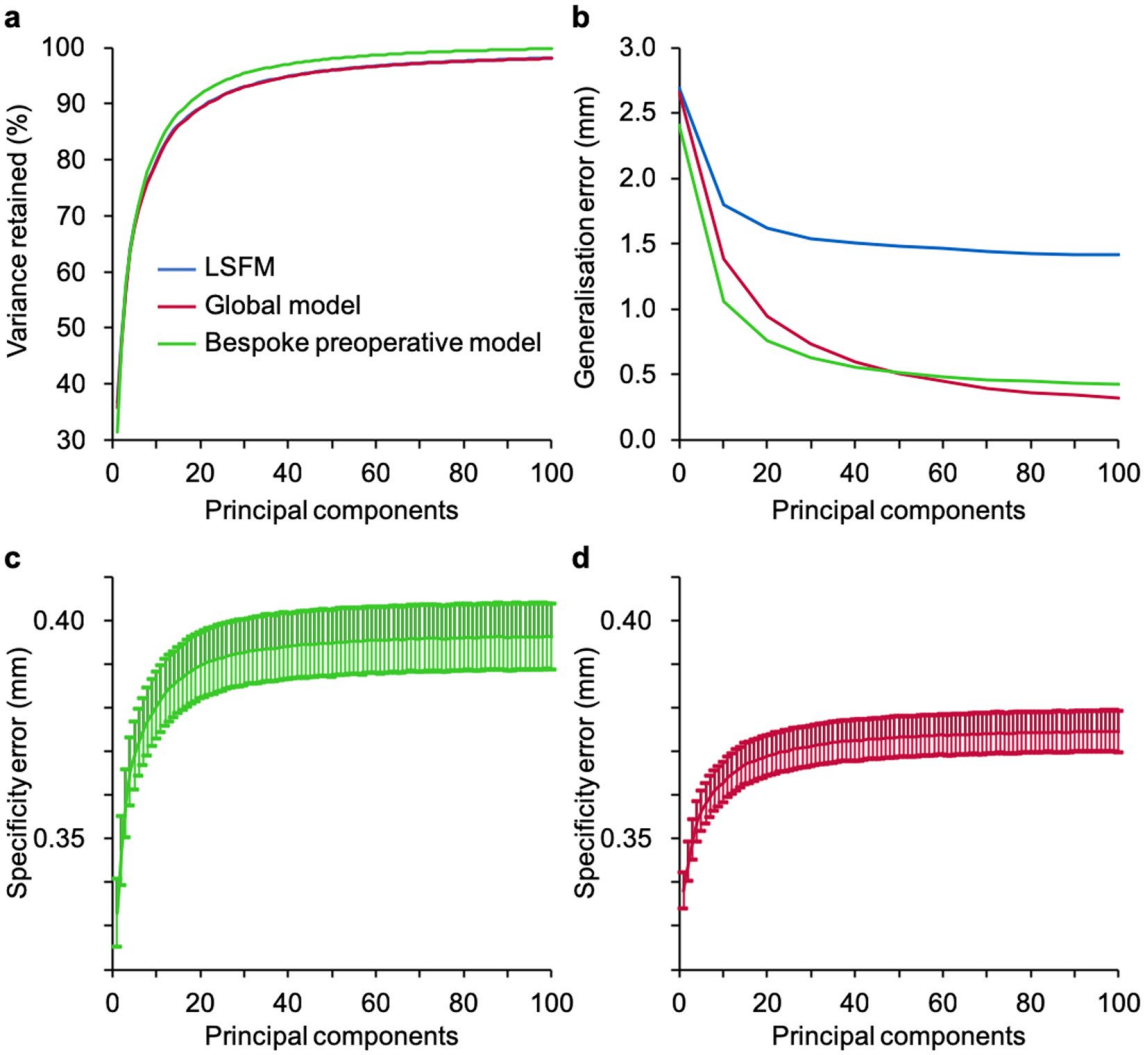
Compactness, generalisation, and specificity for the three models. Characterisation of the three 3DMM compared to the large-scale facial model (LSFM), a state-of-the-art benchmark. (**a**) Compactness (the amount of variance retained for a certain number of principal components) at 10 components amounted to 79.6% for the global model (red), 81.8% for the bespoke preoperative model (green) and 79.9% for LSFM (blue). (**b)** Generalisation (the ability to describe patient faces that were not used to construct the original model) at 100 components equalled to 0.3 mm, 0.4 mm, and 1.4 mm for the global model, bespoke preoperative model, and LSFM, respectively, indicating patient data are required to model patient faces accurately. (**c**) Specificity (how well synthetic faces resemble real faces) for the bespoke preoperative model showed an error of 0.40 ± 0.01 mm, and **d**, specificity for the global model showed an error of 0.37 ± 0.01 mm.

Our models were characterised and validated with the following intrinsic metrics: compactness, generalisation, and specificity (see Methods). Additionally, we benchmarked the performance of our models to the large-scale facial model (LSFM)^29^, a state-of-the-art 3DMM constructed with 9,663 scans. Compactness showed that 81.8% and 91.6% of the variance are respectively described by the first 10 and 20 principal components for the bespoke preoperative model, 79.6% and 89.3% for the global model, and 79.9% and 89.3% for LSFM (Fig. 1a). The generalisation error demonstrated the ability to describe patient faces that were not used for training. Using leave-one-out cross-validation, at 100 components, we found that the global (0.3 mm) and bespoke preoperative (0.4 mm) models outperformed LSFM (1.4 mm), due to lack of patient data in the latter (Fig. 1b). Moreover, the bespoke preoperative model initially outperformed the global model, but after 48 components this trend reversed, as the bespoke model ran out of statistical variance sooner due to a lower number of samples. For specificity, we synthesised faces (n = 10,000) and compared them to their closest real neighbour. Values in the range of 0.3–0.4 mm quantitatively indicated good agreement with real faces (Fig. 1c,d).

### Qualitative and quantitative shape evaluation

**Figure 2:**
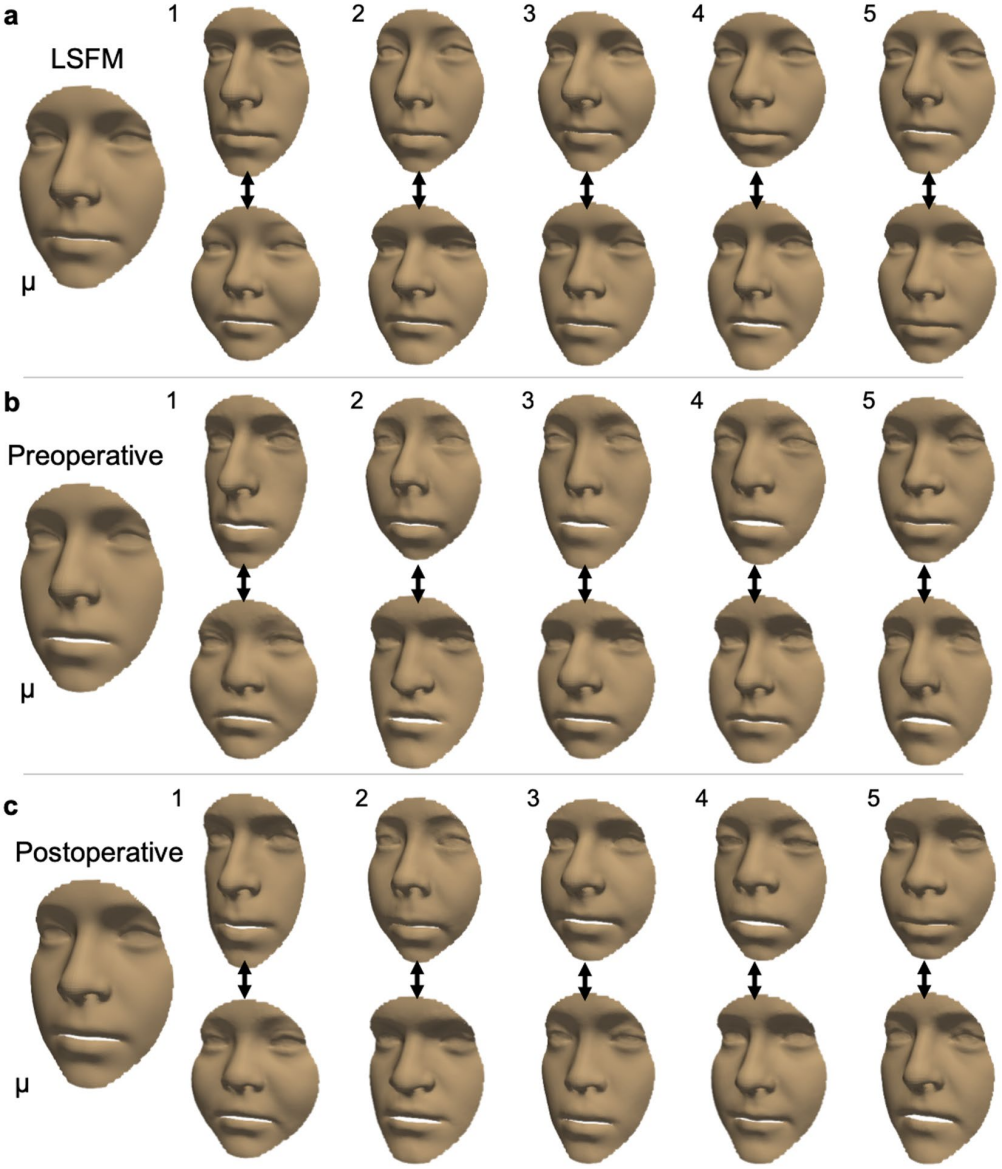
Visualisation of the mean shape and variation for a non-patient face, a preoperative face, and a postoperative face. A qualitative illustration of the mean shape (μ) and first five shape eigenvectors, with weights for the standard deviation (σ_i_) of + 3σ_i_ (top row) and −3σ_i_ (bottom row). (**a**) LSFM represented a non-patient population and acted as a benchmark for our models. (**b)** The bespoke preoperative model, constructed from preoperative 3D scans (n = 119). (**c**) The bespoke postoperative model, constructed from postoperative 3D scans (n = 127).

**Figure 3:**
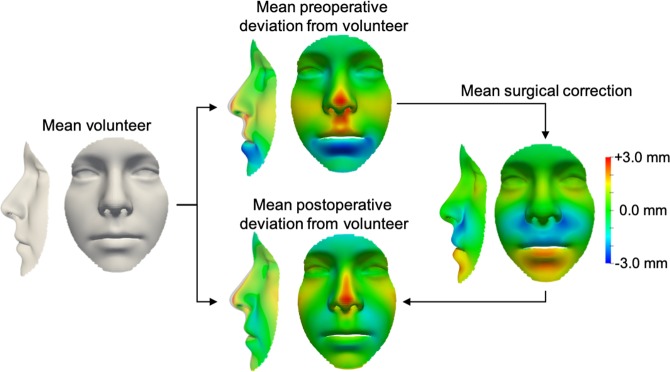
Comparison of the mean non-patient, preoperative, and postoperative face. Colourmaps illustrate deviations from the average volunteer face. The mean preoperative face colourmap is consistent with indications for orthognathic surgery – our cohort of orthognathic patients shows upper jaw underdevelopment (red) and lower jaw overdevelopment (blue). The mean surgical correction appropriately ameliorated jaw underdevelopment or overdevelopment; however, the mean postoperative face retained some preoperative nose shape features.

To investigate how the three models differed, we qualitatively and quantitatively evaluated the shape and variance. Specifically, the mean shape and first five principal components with standard deviation (σ_i_) of +3σ_i_ and −3σ_i_ were computed (see Methods), and differences between the mean shapes were calculated. In the average volunteer face (Fig. 2a) and in the postoperative face (Fig. 2c), lengthening-widening (component 1) and concavity-convexity (component 2) captured most variance, whilst in the mean preoperative face (Fig. 2b), a component of under-overdevelopment of the upper and lower jaw (component 2) was present. These differences were confirmed by a direct comparison of the mean preoperative face to the mean volunteer face (Fig. 3), revealing maxillary hypoplasia (underdevelopment of the upper jaw) and mandibular hyperplasia (overdevelopment of the lower jaw) preoperatively in our patient cohort. The operation successfully ameliorated the jaw discrepancy but some nose orthognathic shape features remained postoperatively (Fig. 3).

### Manifold visualisation

**Figure 4:**
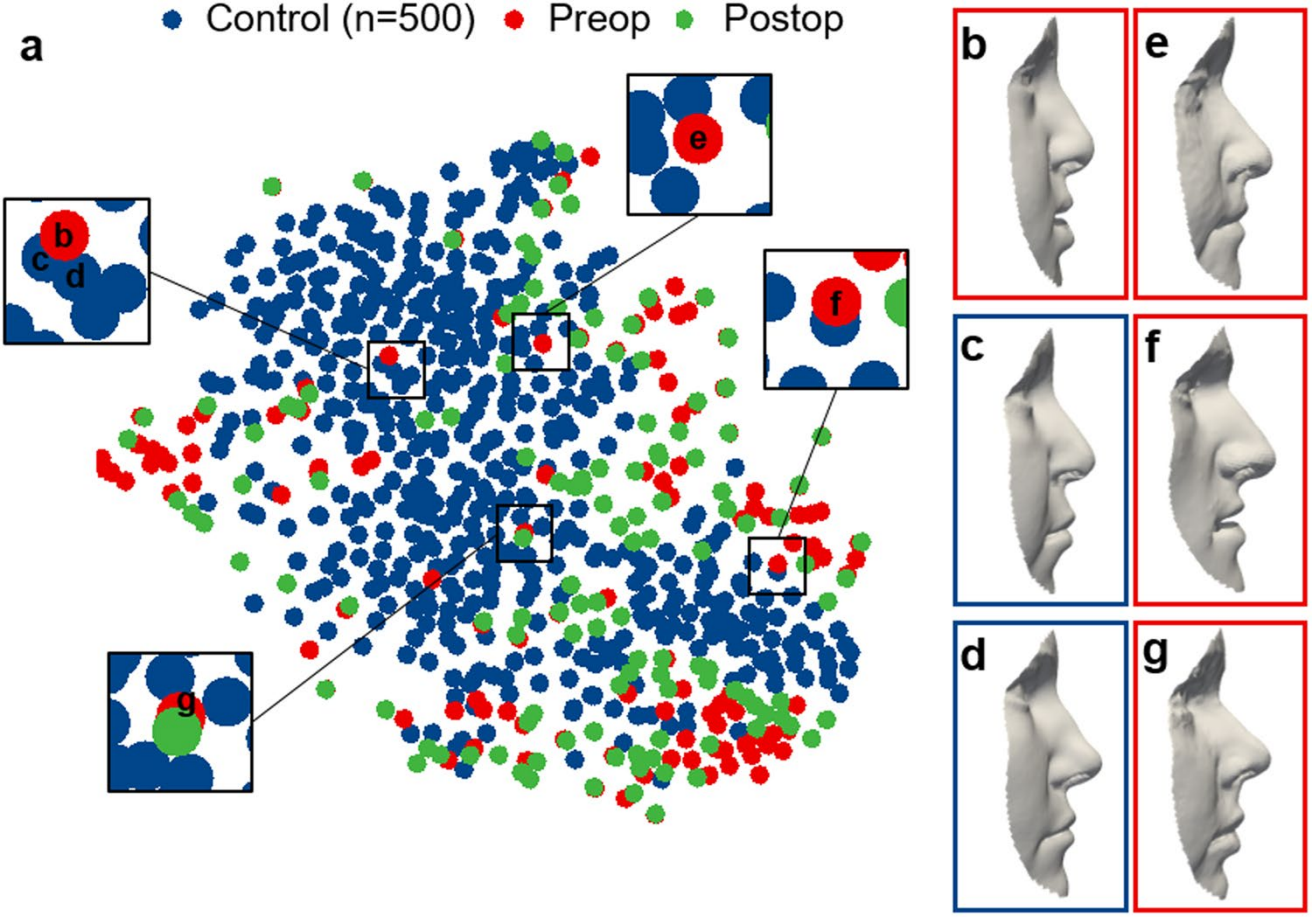
t-SNE embedding of the high-dimensional face manifold. (**a**) The t-SNE embedding in two dimensions was generated with randomly sampled non-patient faces, for visualisation purposes, and labelled according to non-patient (blue, n = 500), preoperative patient (red, n = 119), and postoperative patient (green, n = 127) faces. Lateral views of (**b**) a patient and (**c**,**d)** two close volunteer neighbours to illustrate shape similarity within the t-SNE embedding, particularly in the upper lip angle. (**e,f)** Faces corresponding to false negatives in the classification experiment.

To test the diagnostic potential, we used t-SNE for dimensionality reduction of the high dimensional shape vectors in order to visualise the global manifold in two dimensions with different hyper-parameters (see Methods, Supplementary Fig. 2). With labels for volunteer, preoperative, and postoperative, no distinct groups were uncovered (Fig. 4a). To elucidate similarity amongst neighbouring faces, we display a patient’s face (Fig. 4b) that is close to two volunteer faces (Fig. 4c,d) in the t-SNE embedding, showing resemblance in the facial profile and particularly in the upper lip area. Patient faces that often appeared to be close to the average volunteer face in the classification experiment, as detailed in the next paragraph, are also displayed (Fig. 4e–g). Although the majority of patient faces, preoperatively and postoperatively, appeared to populate the perimeter of the t-SNE embedding, no distinct groups were observed which suggests that patient faces and average volunteer faces, overall, demonstrated substantial shape similarity.

### Classification for diagnosis

**Figure 5:**
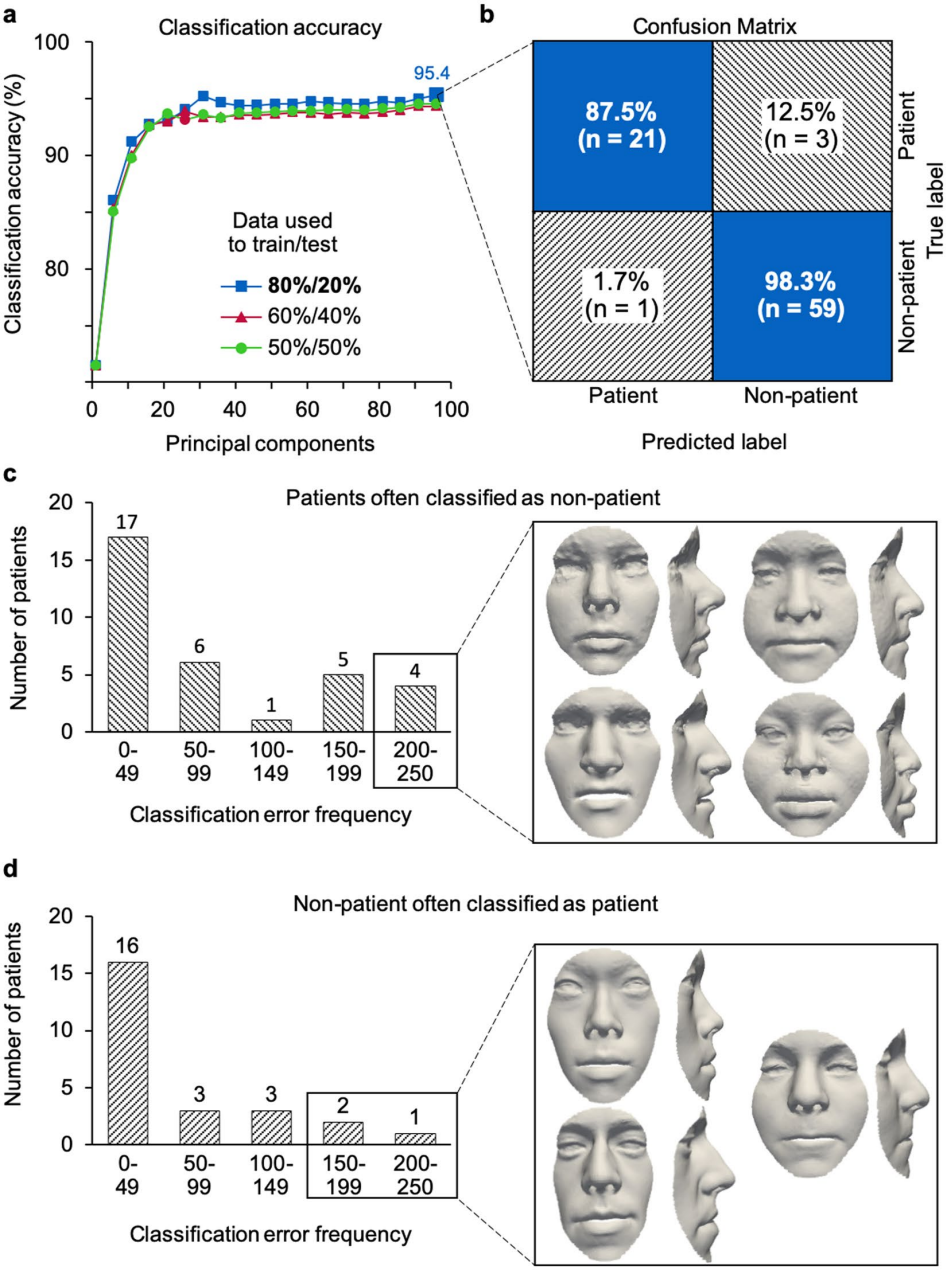
Classification of preoperative patient and non-patient faces. (**a**) A split of 80–20% provides 95.4% classification accuracy at 96 principal components, superior to other splits. (**b)** Average confusion matrix, obtained from classification using preoperative patient scans (n = 140) and randomly selected non-patient scans (n = 280), representing the average of 1,000 iterations. With an 80–20% split, patient (n = 112) and non-patient (n = 224) scans were used for training and patient (n = 28) and non-patient (n = 56) for testing. Orthognathic shape features were diagnosed with 95.5% sensitivity and 95.2% specificity, and with a positive and negative predictive value of 87.5% and 98.3%, respectively. (**c)** For 1,000 iterations, 4 unique patient scans were classified as false positive in more than 200 out of 1,000 iterations, and (**d)** 3 unique volunteer scans were classified as false negative in more than 150 out of 1,000 iterations, suggestive of untreated patients in our volunteer sample.

Classification was performed with all preoperative patient scans (n = 119) and randomly sampled subsets of volunteer face scans in the 14–28 age range (see Methods). Three different splits for training and testing were investigated for 1,000 iterations: a split of 80–20% between training and testing data provided overall classification accuracy of 95.4% (Fig. 5a). Patient faces were diagnosed with 95.5% sensitivity and 95.2% specificity, and a positive and negative predictive value of 87.5% and 98.3%, respectively (Fig. 5b). False negatives – patient faces incorrectly labelled as being from the volunteer sample (Figs 4e–g, 5c) – were observed with a frequency of 12.5% and 4 patients were incorrectly classified in more than 200 out of 1,000 iterations. False positives – volunteer faces incorrectly labelled as patients (Fig. 5d) – were less common with an occurrence of 1.7%, and 3 patients were incorrectly classified in more than 150 out of 1,000 iterations.

### Regression for surgery simulation

**Figure 6:**
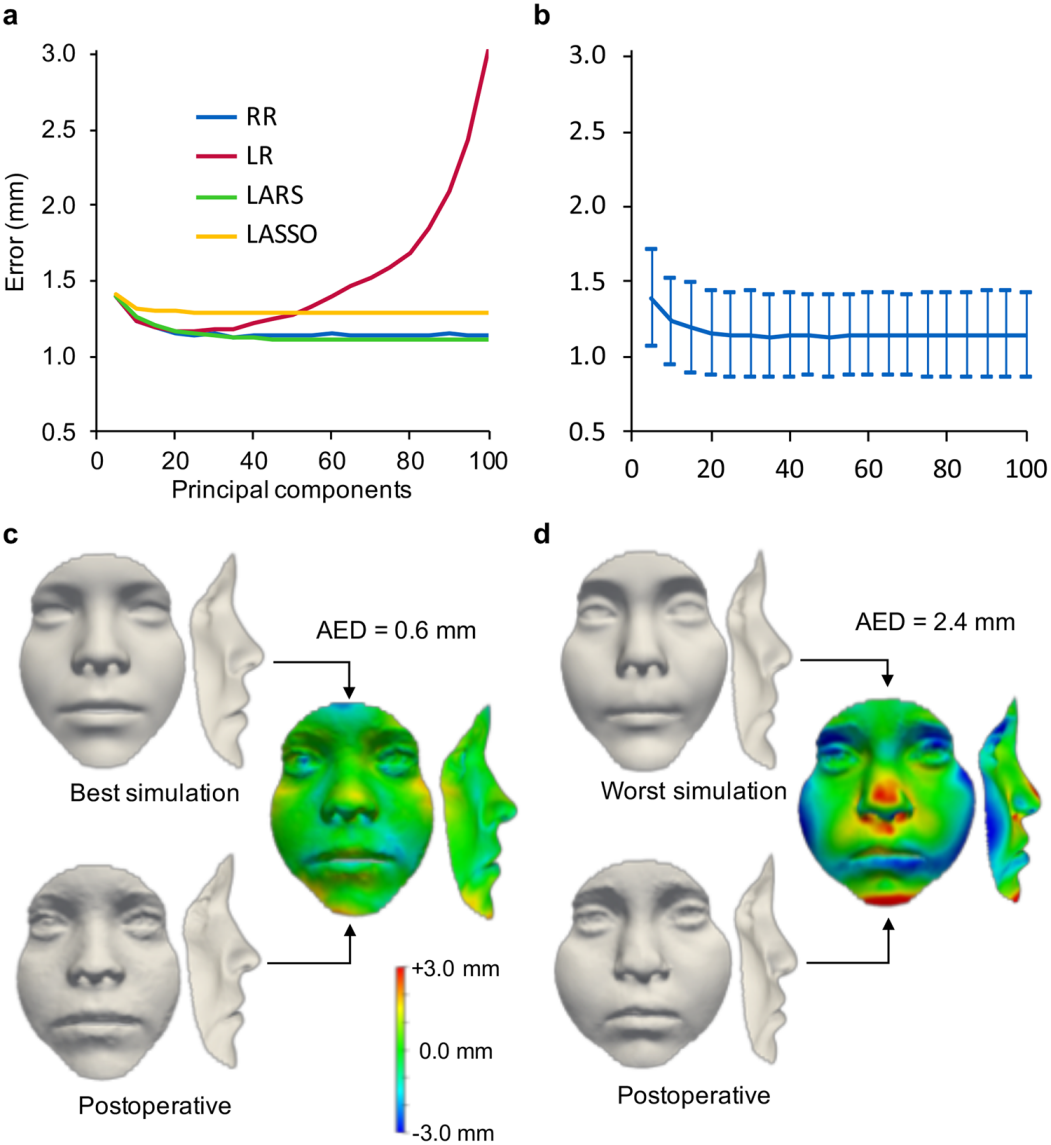
Regression for postoperative face shape simulation. (**a**) Overall error of the ground-truth postoperative face shape compared to the simulated shape using ridge regression (RR), linear regression (LR), least-angle regression (LARS), and least absolute shrinkage and selection operator regression (LASSO). (**b**) Mean and standard deviation for ridge regression: at 100 components, average Euclidean distance = 1.1 mm, s.d. = 0.3 mm. (**c)** The best simulated face, with an error of 0.7 mm between the simulated shape and the postoperative face shape (**d)**. The worst simulated face, with an error of 2.4 mm as compared to the postoperative face shape.

To demonstrate the automated simulation of the postoperative face shape, we tested linear regression (LR), ridge regression (RR), least-angle regression (LARS), and least absolute shrinkage and selection operator regression (LASSO) on our global model (see Methods). The average error between the predicted shape and the ground-truth postoperative shape, at 100 components, was lowest with LARS (1.1 ± 0.3 mm) and RR (1.1 ± 0.3 mm), followed by LASSO (1.3 ± 0.3 mm) and LR (3.0 ± 1.2 mm) (Fig. 6a), which is as accurate as traditional computer-assisted surgical planning methods^23,24^. Using more than 40 components, LR exhibited overfitting which reduced its generalization beyond the training data. To demonstrate the quality of our patient-specific predictions, the differences between preoperative, postoperative and simulated, were visualised and quantified (Fig. 6b). To check that predictions were indeed patient-specific rather than mimicking the population mean, all simulated faces (n = 113) were additionally compared to the mean global face and mean bespoke postoperative face (Supplementary Fig. 3). At 100 components, the difference between RR simulations is much smaller compared to the postoperative 3D scan (1.1 mm, see above) than compared to the mean global face (1.8 mm) and the mean bespoke postoperative face (1.6 mm).

## Discussion

Although there has been great interest in the use of machine learning in plastic and reconstructive surgery, a lack of data and complex interpretability currently limit its adoption in routine clinical practice^36^. In this study, we have introduced a novel approach involving 3DMM trained with 4,216 3D. Using a state-of-the-art computer vision framework^37^, we designed our model that comprehensively integrates high-quality 3D scans to automatically classify orthognathic patient faces and faces from volunteers – as an indication if someone should be seen by a specialist based on their aesthetics – and to automatically predict the patient-specific postoperative outcome. Our model can help objective assessment of preoperative and postoperative face shape, which may help inform patients better during a medical consultation. Additionally, this approach provides a goal-driven surgical planning approach for the surgeon.

Our machine learning approach has several important advantages over computer-assisted surgical planning with traditional software. Conventional surgical simulation is a time-consuming explorative process in which the surgeon manually tests various procedural approaches and assesses the optimal osteotomy and bone position. Our model accurately and automatically predicts the postoperative face shape and reduces the planning process to a single step. However, it does leave the surgeon to decide on the appropriate surgical procedure that delivers the simulated face shape since the surface scans do not contain volumetric bone data. To automatically project the necessary bone movements, a mathematical method that can inversely deduce the modification to the skull to achieve a soft tissue shape^38^, or a combined soft tissue-skeletal model will need to be implemented^39^. However, these models would require a large number of head CT or MR images, thus renouncing the advantages that 3D surface imaging has over volumetric imaging methods. Moreover, large CT and MR image databases are currently not available.

Considering our results, the average models showed an interesting difference between the average volunteer and the postoperative face shape. Whilst the operation successfully ameliorated the jaw discrepancy, some preoperative nose shape features remained postoperatively which is in line with known shape effects of Le Fort I advancement^40–42^. Looking at the classification results, the false negative rate of 12.5% is undesirable as real patients would be missed by the model. We partly attribute this to multifactorial indications for surgery, as previous reports showed that aesthetics is the primary driver for surgery in only 71% of patients^43^. Thus, shape alone may not be the main consideration for at least 12.5% of orthognathic surgery patients. To further improve on the performance of the model, additional scans should be collected, and stricter selection criteria should be adopted to exclude volunteers with mild functional and aesthetic indications for orthognathic surgery.

Indeed, we propose that our model should be used as a machine-learning-based support tool in clinical decision-making, not to fully replace human assessment, and further improvements to the model can be made. Whilst the false positive rate of 1.7% is low, it is substantially larger than the incidence of craniofacial anomalies, including jaw malformation (1 in 1,600 live births^44^) and the incidence of cleft lip and palate (1 in 700 live births, with about 20% requiring an operation later in life^45^). Faces in the LSFM database were collected from the general population where subjects were not excluded for facial anomalies, or untreated functional issues volunteers’ jaws – as this information was not available. It should also be considered that orthognathic surgery has become such a routine practice that some subjects may choose to undergo surgery for mild anomalies that are present within the general population, and no perfect binary classifier exists. The reported accuracy of computer-assisted orthognathic surgery simulation ranges from 0.5 mm to 2.0 mm, depending on the software used^23–26^. Clinically meaningful predictions can be obtained with most commercial software, but intrinsic limitations restrict its use in doctor-patient communication^24^. We demonstrated that our model performs within the range of traditional programs whilst being fully-automated. Although our model shows high sensitivity and specificity, the results also suggest there is scope for further improvements, for example by employing increasingly powerful algorithms and by having access to larger numbers of data.

Recently, in computer vision and machine learning, new modelling frameworks and algorithms have been developed that achieve remarkable success in various applications. Deep Neural Networks (DNNs)^46^, including Convolutional Neural Networks (CNNs)^47^ and Generative Adversarial Networks (GANs) have greatly impacted and increased the performance of automatic systems designed for speech recognition, visual object detection, scene recognition, and face recognition. Whilst it is likely that these models will be used for clinical application in the near future, including computer-assisted surgical planning, their big data requirement limits its current use, and approaches that handle 3D data are still relatively poor compared to more traditional methods like 3DMM. Therefore, the first step to improve further the performance of our models is to increase the number of scans. Generally, machine learning and artificial intelligence rely on big data for their success, but for rare diseases there are limited resources and it is often difficult to obtain access to high-quality, standardised data^36^. Cloud-based platforms have been proposed to integrate data collection, ultimately to improve the quality of care for rare diseases^48^. Alternatively, unsupervised methods have been tested^49^, although their use has not been demonstrated on patient populations. In addition, with the projection of 7.2 billion smartphone subscriptions globally in 2023^50^, and the potential of using smartphones to capture high-quality photos and 3D scans^51^, mobile devices equipped with diagnostic algorithms will play an increasingly important role in low-cost universal care^46^. Our approach, relying on non-ionising 3D scans, can help to accelerate this development and pave the way for shape analysis in other parts of surgery, including craniofacial and aesthetic surgery, and to replace applications that rely on CT scans^52^. A second way of increasing the performance of our model would be the integration of shape data and electronic medical records to create a multimodal machine-learning approach. This could help improve our understanding of how functional and aesthetic indications correlate to various standardised patient outcomes^53^ and for phenotype-genotype correlations^54^.

Clinical 3DMM applications find broad utilisation in plastic and reconstructive surgery^2^, and potentially other fields including facial recognition^33^, expression normalisation^34^, and face reconstruction from video^35^. To support further development in this direction, we have made the LSFM available (https://xip.uclb.com/i/healthcare_tools/LSFM.html).

In summary, we have demonstrated the clinical potential of a large-scale clinical 3DMM, a machine-learning-based framework involving supervised learning, constructed only with non-ionising 3D surface scans. First, automated image processing enables classification, which can provide a binary output whether or not someone should be referred to a specialist. Second, a specialist can automatically simulate the postoperative face shape, which reduces the computer-assisted planning process to a single step. The performance of both classification and regression supports the paradigm of using machine-learning-based tools in clinical decision making and specifically computer-assisted surgical planning. Future validation of the model in larger patient cohorts and diverse surgical specialisms, or multimodal models where shape models are combined with electronic medical records, may lead to valuable new diagnostic and planning tools, ultimately facilitating low-cost care, objective treatment planning and evaluation, and safer and more precise surgery.

## Methods

### Institutional review board statement

Patients provided informed consent and data for were retrospectively retrieved from electronic medical records after receiving approval from the Institutional Review Board at Boston Children’s Hospital (#00019505) and the Human Investigations Committee at Yale-New Haven Hospital (HIC #110100793). Data was analysed in accordance with the guidelines laid out in the Declaration of Helsinki.

### Data sources

Two face databases were used, one database containing faces from the general public and one propriety patient database. Non-patient face scans were collected from the Large Scale Facial Model (LSFM)^37^ database which is available under a non-commercial licence for academic use^55^. LSFM comprises 9,663 3D scans from volunteers taken with a 3dMD face system (3dMD LLC, Atlanta, GA, USA) under standardised imaging conditions at the Science Museum in London with the following demographics (Table 1): mean age = 24.5 ± 14.7 years, 48% male and 52% female, and ethnicity = 82% White, 9% Asian, 5% mixed heritage, 3% Black, and 1% other. The patient database includes 274 3D surface scans taken with the Vectra M3 system (Canfield Scientific, Parsippany, NJ, USA) under standardised conditions, collected from 151 patients who underwent orthognathic surgery at Boston Children’s Hospital and Yale-New Haven Hospital between December 2010 and September 2017 (Table 1), with the following demographics: mean age = 18.4 ± 2.4 years, 44% male and 56% female, and ethnicity = 76% White, 10% Asian, 10% Mixed Heritage/Other, and 8% Black. Non-syndromic patients who had a Le Fort I or bimax osteotomy were included in this retrospective study providing they had complete medical records including preoperative and long-term postoperative 3D surface scans. Additional information that was extracted from the electronic medical records including scan date, operation date, surgical procedure, indication for surgery, and syndromic diagnosis. The patient dataset analysed in this study is not publicly available due to ethical restrictions.

### 3D morphable model construction

Our 3DMM training pipeline, based on the approach proposed in^29^, operates in four main functional blocks:

1. Automatic annotation – each 3D mesh was rendered from a number of virtual cameras positioned around the subject into 2D images. A vector defines the geometry of each 3D facial mesh: ${\bf{X}}={[{{\bf{x}}}_{1}^{T},{{\bf{x}}}_{2}^{T},\ldots ,{{\bf{x}}}_{n}^{T}]}^{T}\in {{\mathbb{R}}}^{3n}$, where *n* is the number of vertices and ${{\bf{x}}}_{i}={[{x}_{x}^{i},{x}_{y}^{i},{x}_{z}^{i}]}^{T}\in {{\mathbb{R}}}^{3}$ describes the X, Y and Z coordinates of the *i*-th vertex. A landmark localisation algorithm – an active appearance model (AAM) – was applied to find the 2D landmarks on the rendered images, and each 2D landmark set was projected onto the 3D surface, rendering the 3D landmarks.
2. Alignment and statistical modelling – the collection of scans was brought into the same space by removing similarity effects (rotation, translation, scale) via generalised Procrustes analysis (GPA); leaving only shape information.
3. Dense correspondence – the aligned collection of 3D scans was registered into a form where each scan had the same number of points joined into a triangulation shared across all scans. Dense correspondence was accomplished via non-rigid ICP (NICP)^56^, using the LSFM mean face as a template. This process deforms the template mesh to the shape of each patient face to obtain a set of deformed templates.
4. Statistical analysis **–** the 3DMM model was built by applying principal component analysis (PCA) on the corresponding meshes and finding the eigenvectors-bases with the greatest variance. Any 3D face shape can be defined as a linear combination of these bases, which makes up the 3DMM as follows:

1 $${{\bf{X}}}^{\ast }={\boldsymbol{M}}+\mathop{\sum }\limits_{i=1}^{d}{\alpha }_{i}{{\boldsymbol{U}}}_{i}={\boldsymbol{M}}+U{\boldsymbol{\alpha }}$$

Where ${\boldsymbol{M}}\in {{\mathbb{R}}}^{3n}$ represents the mean shape, ${\bf{U}}=[{{\boldsymbol{U}}}_{1}\ldots {{\boldsymbol{U}}}_{{\boldsymbol{d}}}]\in {{\mathbb{R}}}^{3{\rm{n}}\times d}$ is the orthonormal basis matrix with columns containing the shape eigenvectors ${{\boldsymbol{U}}}_{i}$, ${\boldsymbol{\alpha }}=[{\alpha }_{1},\ldots ,{\alpha }_{d}]\in {{\mathbb{R}}}^{d}$ is the shape vector containing the coefficients that define a specific shape instance for a given deformable shape model. Any given input face mesh ${\bf{X}}$ can be projected on the model subspace by identifying the shape vector ${\boldsymbol{\alpha }}$ that produces a shape as close as possible to ${\bf{X}}$. The optimal shape vector and projection ${\rm{P}}({\bf{X}})$ are given by:

2 $${\boldsymbol{\alpha }}={{\bf{U}}}^{T}({\bf{X}}-{\boldsymbol{M}}),{\rm{P}}({\bf{X}})={\boldsymbol{M}}+{\bf{U}}{{\bf{U}}}^{{\rm{T}}}({\bf{X}}-{\boldsymbol{M}})$$

### Model characteristics

The intrinsic characteristics of each 3DMM were evaluated, including compactness, generalisation, and specificity^57^. Compactness is a measure of the cumulative variance in the data that is retained with a certain number of principal components and was extracted from the model construction. Generalisation describes how well a face unknown to the 3DMM can be approximated by the existing model. Specifically, we used leave-one-out cross-validation for all patient faces in all three models (LSFM, global, bespoke preoperative): a model was constructed for all faces but one, and then fitted to the excluded face. The error between the excluded face and the model was quantified using the average Euclidean distance, with a large generalisation error suggesting overfitting, the inability of a model to represent previously unseen faces. We repeated this for all patient faces. Specificity measures how well synthesised faces can be approximated by ground-truth images. Specifically, faces (n = 10,000) were randomly synthesised for each model, and the specificity error was computed as the lowest average Euclidean distance of all vertices between a synthesised face and the closest ground-truth neighbour.

### Error quantification

The error was quantified using the average Euclidean distance (AED), calculated from the per-vertex distance between two meshes:

$$AED=\frac{{\sum }_{i=1}^{n}\sqrt{{({x}_{i,A}-{x}_{i,B})}^{2}+{({y}_{i,A}-{y}_{i,B})}^{2}+{({{\rm{z}}}_{{\rm{i}},{\rm{A}}}-{z}_{i,B})}^{2}}}{n}$$

where x, y, and z corresponded to the Cartesian coordinates of mesh *A* and *B*, and *n* was the number of vertices per mesh. Each mesh was in dense correspondence and therefore vertex *i* represented the same anatomical location in both meshes. To compute signed errors (such as shown in Figs 3 and 6), the error was positive if the reference mesh had a larger value on the z-axis.

### Manifold visualisation

T-distributed stochastic neighbour embedding (t-SNE) was used as a dimensionality reduction technique^58^ to visualise a high-dimensional manifold onto a 2-dimensional space. We tested various hyper-parameters (perplexity = 2 to 100; iterations = 1,000 to 5,000, Supplementary Fig. 2) as well as different numbers of randomly sampled non-patient faces (n = 200 to n = 3,000) together with all preoperative patient faces (n = 119) and postoperative patient faces (n = 127).

### Classification

Classification was performed using a subgroup of randomly selected volunteer faces (n = 300) and faces from pre-operative patients (n = 119). We split the whole dataset in a stratified manner with various proportions between training and test set (80–20%, 60–40%, and 50–50%). Thus, for the 80–20% case, we used patient (n = 95) and non-patient (n = 240) faces for the training set and patient (n = 24) and non-patient (n = 60) faces for the test set. For the classifier, we chose a Support Vector Machine (SVM)^59^ with linear kernel $x,x^{\prime}$ as SVMs are powerful tools for small sample size problems. We employed the scikit^60^ implementation of SVM with “one-vs-the-rest” multi-class strategy with default values for the penalty parameter (*C* = 1.0) and gamma. To calculate the mean accuracy, training and test sets were created according to a Monte-Carlo cross-validation scheme by randomly selecting the training and test set 1,000 times.

### Regression

To automatically predict face shape outcomes based on the preoperative scan, linear regression (LR), ridge regression (RR), least-angle regression (LARS), and least absolute shrinkage and selection operator regression (LASSO) were tested, using their scikit^60^ implementation. For RR and LASSO, the alpha parameter that defines the strength of regularization term was set to 0.5 and 0.1, respectively. For LARS, the number of nonzero coefficient was set to 1. We kept the default values for all the other parameters. We used the global model to perform regression, which included volunteer (n = 3,664), preoperative (n = 113) and for the same unique patients their postoperative scans postoperative (n = 113) faces. For our experiment, we used the leave-one-out scheme. A design matrix was learnt between the components of the preoperative and postoperative patients, which was then used to map the preoperative components to the postoperative components. We repeated the experiment leaving out each patient and for various components (113 times). All regression methods but LR penalised the weight of the components with a regulizer which made them more robust to overfitting.

## Supplementary information

Supplementary information
