# Supplementary material for: A machine learning framework for automated diagnosis and computer-assisted planning in plastic and reconstructive surgery
Source: Sci Rep. 2019 Sep 19;9:13597. doi: 10.1038/s41598-019-49506-1 (PMC6753131; doi:10.1038/s41598-019-49506-1)
Supplement: Supplementary file 1 — Supplementary information [file 41598_2019_49506_MOESM1_ESM.pdf]

SUPPLEMENTARY INFORMATION

A machine learning framework for automated diagnosis and computer-assisted planning in plastic and reconstructive surgery

Paul G.M. Knoops[1,2,†], Athanasios Papaioannou[1,3,†], Alessandro Borghi[1], Richard. W.F. Breakey[1], Alexander T. Wilson[4], Owase Jeelani[1], Stefanos Zafeiriou[3], Derek Steinbacher[4], Bonnie L. Padwa[2], David J. Dunaway[1], Silvia Schievano[1,]*

1. UCL Great Ormond Street Institute of Child Health, London, UK & Craniofacial Unit, Great Ormond Street Hospital for Children, London, UK
2. Department of Plastic and Oral Surgery, Boston Children's Hospital & Harvard School of Dental Medicine, Boston, MA, USA
3. Department of Computing, Imperial College London, London, UK
4. Department of Plastic and Reconstructive Surgery, Yale University School of Medicine, New Haven, CT, USA

† These authors contributed equally to this work.

* Email: s.schievano@ucl.ac.uk

Supplementary figures

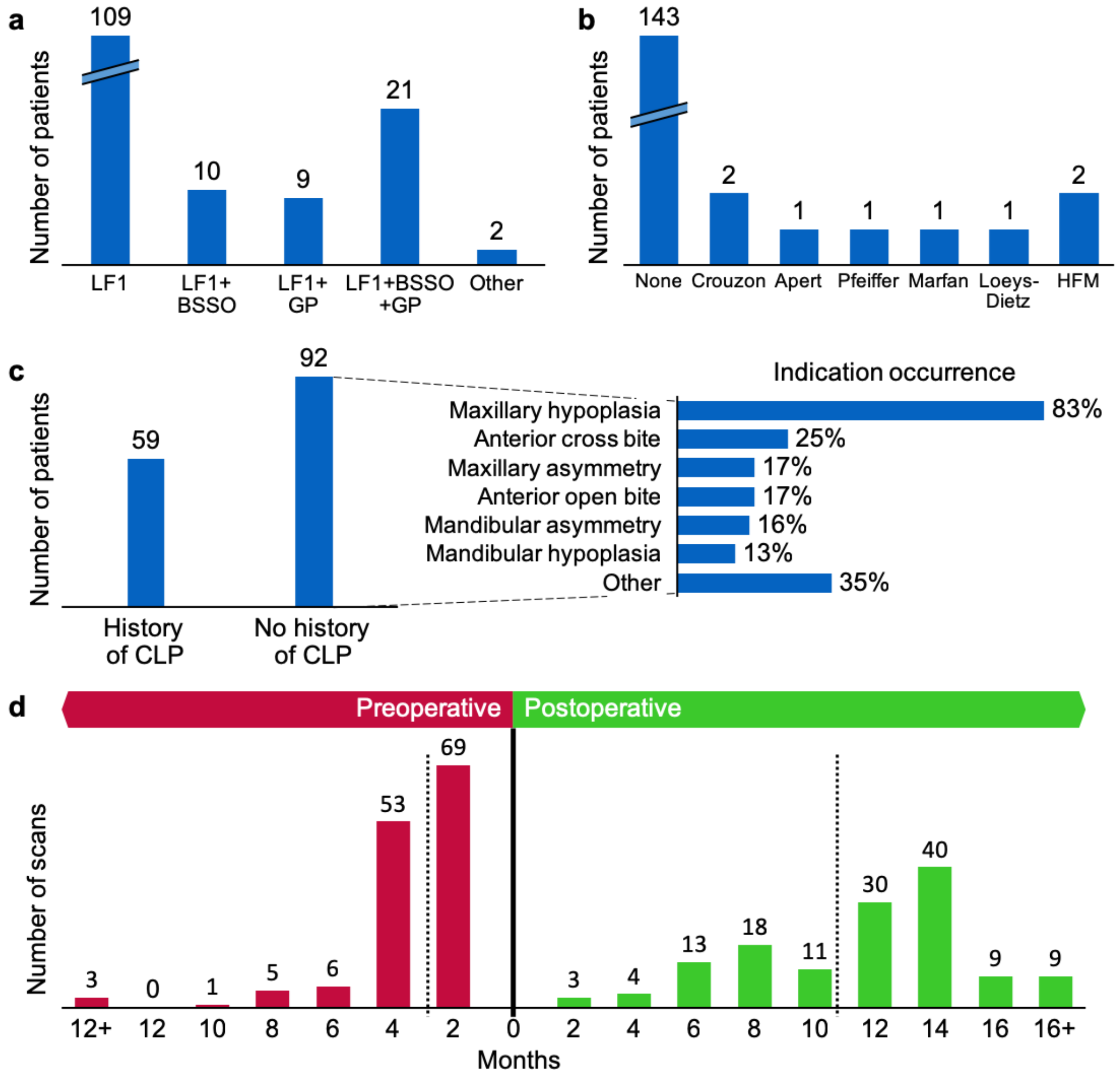

Supplementary Fig. 1 Patient characteristics. a, Number of patients per surgical procedures, LF1: Le Fort 1 osteotomy, BSSO: bilateral split osteotomy, GP: genioplasty. **b**, Number of patients with syndromic diagnosis, HFM: hemifacial microsomia. **c**, Number of patients according to the main indication for surgery: history or no history cleft lip and/or palate (CLP). For those without a history of CLP, often multiple indications are present. **d**, Timing of preoperative and postoperative scans: average preoperative scan was 73 ± 86 days before surgery (red, dotted line) and the average postoperative scan 326 ± 123 days (green, dotted line) after surgery.

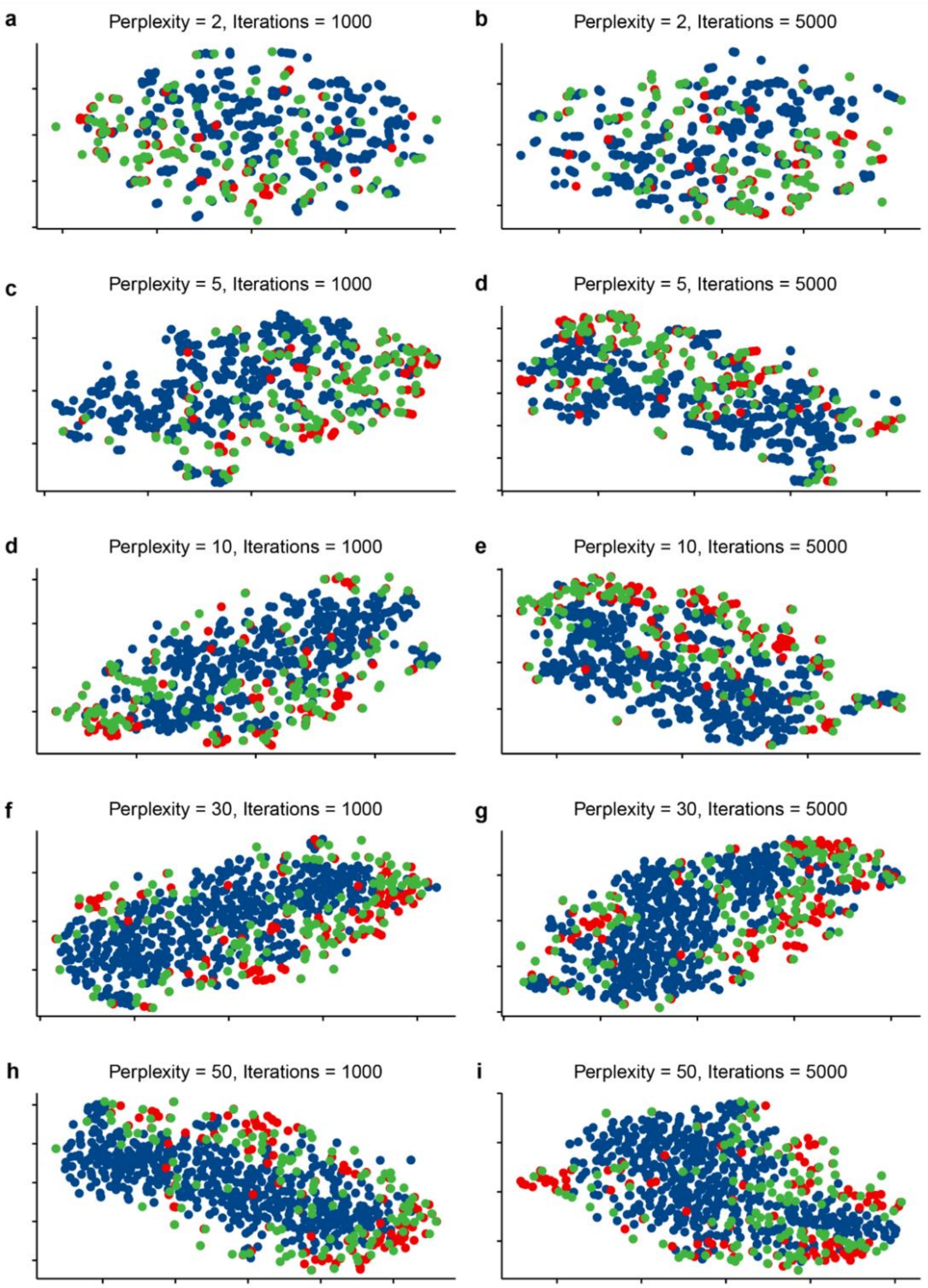

Supplementary Fig. 2 Hyper-parameters settings for t-SNE embedding. Different visualisations for the manifold including non-patient (blue, n=500), preoperative patient (red, n=140) and postoperative patient (green, n=133) faces for perplexity = 2, 5, 10, 30, 50 and iterations = 1,000, 5,000.

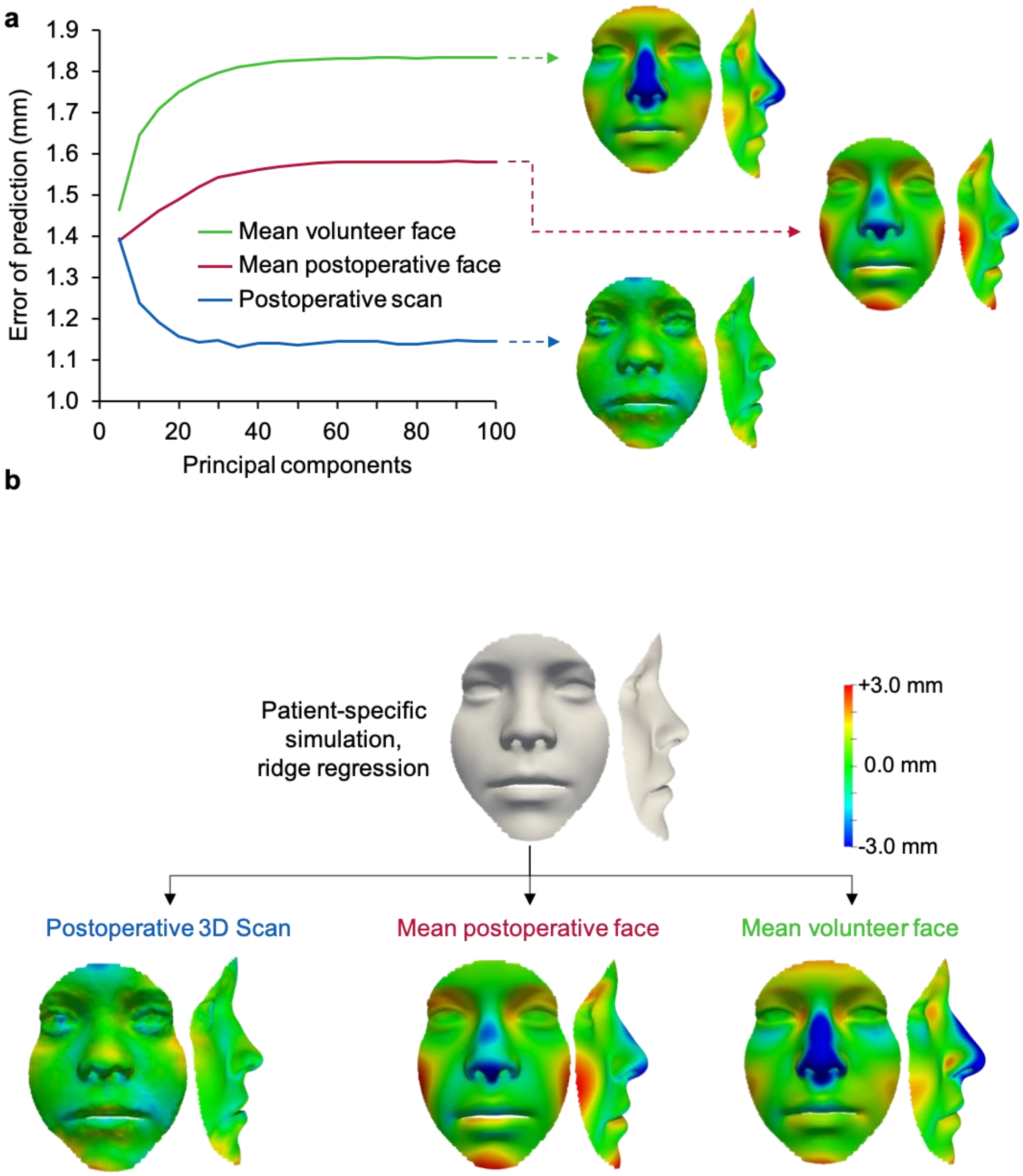

Supplementary Fig. 3 Error evaluation. a, Colourmaps illustrate that the patient-specific simulations are better than predicting a face closer to the mean volunteer or the mean postoperative face; a comparison of error between the postoperative 3D scan (n=127) and patient-specific ridge regression simulations (1.1 ± 0.3 mm), the average postoperative face (1.6 ± 0.5 mm), and the average non-patient face (1.8 ± 0.5 mm). **b**, Colourmaps elucidate how the best patient-specific simulation compares to the ground-truth postoperative face, the mean postoperative face, and the mean volunteer face.
